# Radiation Oncology Fellowship: a Value-Based Assessment Among Graduates of a Mature Program

**DOI:** 10.1007/s13187-020-01767-5

**Published:** 2020-07-18

**Authors:** Emma Ito, Fabio Y. Moraes, Matthew Ramotar, Isis Lunsky, Hany Soliman, Charles N. Catton, Zahra Kassam, Gerard Morton, Sarah Tosoni, Mary Gospodarowicz, Rebecca K.S. Wong, Fei-Fei Liu, Peter W. M. Chung

**Affiliations:** 1 Radiation Medicine Program, Princess Margaret Cancer Centre, University Health Network, 610 University Avenue, Toronto, M5G 2M9 Canada; 2 Department of Oncology, Queen’s University, Kingston, Ontario Canada; 3 Department of Radiation Oncology, University of Toronto, Toronto, Ontario Canada; 4 Department of Radiation Oncology, Odette Cancer Centre, Sunnybrook Health Sciences Centre, Toronto, Ontario Canada; 5 Stronach Regional Cancer Centre, Newmarket, Ontario Canada

**Keywords:** Radiation Oncology, Fellowship, Medical education

## Abstract

The University of Toronto – Department of Radiation Oncology (UTDRO) has had a well-established Fellowship Program for over 20 years. An assessment of its graduates was conducted to evaluate training experience and perceived impact on professional development. Graduates of the UTDRO Fellowship Program between 1991 and 2015 were the focus of our review. Current employment status was collected using online tools. A study-specific web-based questionnaire was distributed to 263/293 graduates for whom active e-mails were identified; questions focused on training experience, and impact on career progression and academic productivity. As a surrogate measure for the impact of UTDRO Fellowship training, a comparison of current employment and scholarly activities of individuals who obtained their Fellow of the Royal College of Physicians of Canada (FRCPC) designation in Radiation Oncology between 2000 and 2012, with (*n* = 57) or without (*n* = 230) UTDRO Fellowship training, was conducted. Almost all UTDRO Fellowship graduates were employed as staff radiation oncologists (291/293), and most of those employed were associated with additional academic (130/293), research (53/293), or leadership (68/293) appointments. Thirty-eight percent (101/263) of alumni responded to the online survey. The top two reasons for completing the Fellowship were to gain specific clinical expertise and exposure to research opportunities. Respondents were very satisfied with their training experience, and the vast majority (99%) would recommend the program to others. Most (96%) felt that completing the Fellowship was beneficial to their career development. University of Toronto, Department of Radiation Oncology Fellowship alumni were more likely to hold university, research, and leadership appointments, and author significantly more publications than those with FRCPC designation without fellowship training from UTDRO. The UTDRO Fellowship Program has been successful since its inception, with the majority of graduates reporting positive training experiences, benefits to scholarly output, and professional development for their post-fellowship careers. Key features that would optimize the fellowship experience and its long-term impact on trainees were also identified.

**Electronic supplementary material:**

The online version of this article (10.1007/s13187-020-01767-5) contains supplementary material, which is available to authorized users.

## Introduction

Cancer incidence rates are increasing worldwide, with more than 50% of all cancer patients requiring radiation therapy [1]. As such, radiation oncology (RO) continues to be a growing specialty. In recent years, a steady increase in the number of programs offering RO fellowship training and the number of clinical RO fellows has been observed [2, 3]. Several studies have reported that an increasing proportion of RO residents are extending their training by pursuing post-residency fellowships to enhance clinical and research competencies, as well as to increase their competitiveness in the challenging job market and academic environment [2, 4–6]. RO fellowships, typically spanning 1 to 2 years, offer opportunities to gain subspecialty expertise and skills in specialized technologies and techniques (e.g., brachytherapy, stereotactic radiation therapy, image guided techniques, proton therapy); pursue research projects with potential for high impact academic publication; and network and build collaborations.

The Radiation Oncology Fellowship Program at the University of Toronto – Department of Radiation Oncology is one of the largest of its kind in the world (https://www.radonc.utoronto.ca). Formally established as an independent department in 1991, the Fellowship Program is open to those who have recently completed specialty training in Radiation Oncology in their home country. The program annually attracts a large number of high caliber candidates from across the world, including Asia-Pacific countries, Europe, Africa, and the Americas. Successful candidates are selected through a competitive process with approximately twice the number of applicants versus fellows accepted. Fellows enroll in Clinical Research Fellowships (approximately 1–2 years) at either the Princess Margaret Cancer Centre (University Health Network) or Odette Cancer Centre (Sunnybrook Health Sciences Centre), both offering rich on-site clinical and research training experiences. As a university-based program for certified specialists that is not accredited by the Royal College of Physicians and Surgeons of Canada (Royal College), a structured program-level assessment of student learning and training experience has not been conducted to date.

This study represents the first effort to formally evaluate the impact of post-residency fellowship training on the careers of its graduates at a single institution. Our objectives were to (1) describe the characteristics of learners who enrolled in the UTDRO Fellowship Program; (2) examine the trends in current employment status and academic productivity of those completing the UTDRO Fellowship, (3) explore the potential impact on academic productivity by evaluating similar metrics in a non-UTDRO trained cohort, and (4) to explore the strengths and weaknesses of our program as recalled by learners through a study-specific survey. We hypothesized that completion of post-residency fellowship training has a positive impact on graduates’ academic output and overall career trajectory. Our findings may help to establish a framework for evaluating the effectiveness of fellowship programs, and to identify key features that would optimize the fellowship experience and its long-term impact on trainees.

## Methods and Materials

Three methods were employed to address our study objectives (Appendix I). First, a retrospective analysis of employment and scholarly activities of individuals who enrolled in the UTDRO Fellowship Program since its formal inception was conducted. Second, a study-specific survey to capture graduates’ perceptions of their fellowship experiences and impact on career trajectory was sent to alumni. Third, a comparator cohort was identified to further characterize the potential effect of UTDRO Fellowship training on employment and academic productivity post-residency.

A waiver of consent was granted for this study by the Research Ethics Board at the University Health Network through an expedited review procedure.

### Study Population

#### Retrospective Cohort

Graduates of the UTDRO Fellowship Program with an enrollment start date between 1991 and 2015 were identified (*n* = 293) from departmental records and the Postgraduate Web Evaluation and Registration system hosted by the Faculty of Medicine at the University of Toronto.

#### Survey Cohort

University of Toronto, Department of Radiation Oncology Fellowship alumni for whom active e-mails were available (*n* = 263) were sent the UTDRO Fellowship Experience Survey as part of a comprehensive approach for quality improvement and impact assessment.

#### Comparator Cohort

Since UTDRO graduates were predominantly from Canada, Australia and the UK, we attempted to identify non-UTDRO trained peers with recognized RO specialty training/certification within these countries. Our search was limited to RO specialists within Canada, as we were only able to access the public directory of the Royal College of Physicians and Surgeons of Canada.

To characterize the employment and scholarly activities of a contemporary cohort of Canadian RO specialists (minimum of 5 years post-residency), individuals who obtained their Fellow of the Royal College of Physicians of Canada (FRCPC) in Radiation Oncology between the years of 2000 and 2012 (*n* = 287) were identified. Specialty recipients were further divided into two subgroups: UTDRO Fellowship alumni (*n* = 57) and others (*n* = 230).

### Data Collection

#### Employment Status and Academic Productivity

An extensive online search of the World Wide Web (Internet) was conducted from May to June 2018 to identify the current country of employment and position(s), as well as academic output and professional (radiation oncology-specific) leadership appointments attained by individuals within the retrospective and comparator cohorts. Multiple online databases were utilized (described in Appendix II) to determine the total number of peer-reviewed articles published by individuals since receiving their FRCPC designation, as well as the number of clinical trials in which individuals were involved as principal investigator or co-investigator.

#### UTDRO Fellowship Experience Survey

A 51-question web-based Google Form survey comprised of multiple choice and open-ended questions was sent electronically to the survey cohort. The questionnaire was divided into three sections: (i) the UTDRO Fellowship experience, (ii) career after the UTDRO Fellowship, and (iii) an evaluation of the Fellowship Program. The survey, which required approximately 15 min to complete, contained questions focused on the individual’s experience of the program, how it influenced their career progression and academic productivity post-fellowship, as well as suggestions for programmatic improvement. Individuals were sent a secure explanatory e-mail describing the objective of the project, voluntary nature of participation, and anonymity of the data collection process, as well as a link to access the online survey. The survey was available for completion over a 14-week period (September–December 2017), over which time six e-mail reminders were sent to non-responders. Anonymized, aggregate responses from completed surveys were compiled in an Excel database.

### Qualitative Analysis

Thematic analysis [7, 8] was conducted on all open-ended survey responses using an inductive approach to identify common shared responses across participants. Responses were grouped based on similar features and classified into common themes which were then ranked from most-to-least mentioned. To ensure inter-rater reliability, two members of the research team conducted analyses independently, with regular meetings to confirm consensus.

### Statistical Analysis

Descriptive statistics were used to summarize the quantitative data and describe survey responses. A *p* value of ≤ 0.05 (Mann-Whitney *U* test) was utilized as the threshold for statistical significance.

## Results

### Demographics of UTDRO Fellows

Between the years of 1991 and 2015, 293 individuals were enrolled in the UTDRO Fellowship Program (Table 1). The median number of fellows accepted annually into the program was 11 (range 1–25). Countries of citizenship were documented for 290 individuals (Appendix III). The majority of fellows were from Canada (29%), followed by Australia (18%) or the UK (16%). Approximately 4% of fellows were from lower-middle-income countries (LMICs), including Ghana (0.3%) and India (4%).

**Table 1:** Demographics of UTDRO Fellows (1991–2015)

		No.	Percent
Year Fellowship started (*n* = 293)	1991–1995	16	(5)
	1996–2000	29	(10)
	2001–2005	57	(20)
	2006–2010	95	(32)
	2011–2015	96	(33)
Gender (*n* = 293)	Female	132	(45)
	Male	161	(55)
Country of citizenship (*n* = 290)	Australia	53	(18)
	Canada	83	(29)
	India	12	(4)
	Ireland	20	(7)
	New Zealand	10	(3)
	UK	46	(16)
	Other	66	(23)
Country of employment (*n* = 293)	Australia	56	(19)
	Canada	100	(34)
	Ireland	12	(4)
	New Zealand	11	(4)
	UK	43	(15)
	USA	12	(4)
	Other	59	(20)
Appointment (*n* = 293)	Leadership	68	(23)
	Research	53	(18)
	Staff oncologist	291	(99)
	University faculty	130	(44)
	Other (e.g., trainee, non-practicing hospital administrator)	2	(1)

Since fellowship completion, most alumni were working in Canada (34%), Australia (19%), or the UK (15%). Almost all alumni were employed as staff radiation oncologists (99%), and held university faculty appointments (44%), research positions (18%), and/or additional leadership roles (23%) in clinical, research, or educational programs, as well as regional, national, or international committees and organizations.

### Demographics of Survey Respondents

The UTDRO Fellowship Experience Survey was completed by 38% (101/263) of the survey cohort. Survey respondents (Table 2) were generally more recent alumni compared with the retrospective cohort (Table 1), but had similar distributions in terms of current country of employment and proportion of those hired as staff oncologists. The majority were graduates of Canadian (22%), UK (22%), or Australian (14%) medical schools. Eighty-nine percent of respondents began their fellowship in 2001 or later, with median fellowship duration being 12 months (range 5–64); the majority (52%) completed their studies between 10 and 14 months. The minimum length of training for fellows was 6 months as defined by the university’s Postgraduate Medical Education Office, although the majority of fellows were enrolled in a 12-month program. Occasionally, fellows departed early due to receiving offers for full-time employment prior to the projected end-date of their fellowship. Even so, these fellows were present long enough to achieve their intended goals, and thus were classified as graduates. Most respondents (57%) were between 30 and 34 years old at the start of their fellowship, and majority had relocated with their family (60%). Eighty-five percent of respondents elected to enroll in the 1-year Fellowship Program and 89% completed their fellowship at the Princess Margaret Cancer Centre.

**Table 2:** Demographics of survey respondents (*n* = 101)

		No.	Percent
Country of medical school training (top 5)			
	Canada	22	(22)
	UK	22	(22)
	Australia	14	(14)
	India	6	(6)
	New Zealand	6	(6)
	Other	31	(30)
Country of radiation oncology training (top 5)			
	Canada	29	(28)
	UK	22	(22)
	Australia	18	(18)
	Chile	5	(5)
	Switzerland	5	(5)
	Other	22	(22)
Year of Fellowship start			
	1991–1995	2	(2)
	1996–2000	9	(9)
	2001–2005	22	(22)
	2006–2010	26	(26)
	2011–2015	42	(41)
Fellowship duration (months)			
	5–9	5	(5)
	10–14	53	(52)
	15–19	15	(15)
	20–24	19	(19)
	25–29	1	(1)
	≥30	8	(8)
Age at start of Fellowship			
	25–29 years old	12	(12)
	30–34 years old	58	(57)
	35–39 years old	26	(26)
	> 40 years old	5	(5)
Relocated with family			
	Yes	61	(60)
	No	40	(40)
Fellowship location			
	Odette Cancer Centre	11	(11)
	Princess Margaret Cancer Centre	90	(89)
Fellowship type			
	1-Year clinical fellowship (80% clinical)	31	(31)
	1-Year clinical research fellowship (50% clinical and 50% research)	55	(54)
	2-Year research fellowship (80% research)	15	(15)

Number of survey respondents per question: 101

### Fellowship Objectives and Scholarly Output of Survey Respondents

The top objectives (Table 7), in order of importance, for pursuing a fellowship at UTDRO were to (1) gain specific clinical expertise, (2) gain exposure to research opportunities with potential to publish, (3) obtain another perspective of radiation oncology in a large academic department, (4) network and build collaborations with world-class academics, and (5) become more competitive in the job market. Almost all (99%) of the respondents believed that the UTDRO Fellowship had mostly or completely fulfilled these objectives (Table 3). Thematic analyses revealed that the top reasons the fellowship program fulfilled the alum’s objectives were (1) clinical experience, (2) research opportunities, (3) networking, (4) mentorship, and (5) that it led to employment (Table 7).

**Table 3:** Fellowship objectives and academic output

		No.	Percent
Additional degrees obtained during Fellowship			
	Master’s Degree	10	(10)
	PhD	4	(4)
	None	87	(86)
Number of peer-reviewed manuscripts resulting from Fellowship (first author or co-author)			
	0	13	(13)
	1–3	54	(53)
	4–6	19	(19)
	7–9	5	(5)
	≥ 10	10	(10)
Number of abstracts resulting from Fellowship (first author or co-author)			
	0	8	(8)
	1–3	54	(53)
	4–6	19	(19)
	7–9	7	(7)
	≥ 10	13	(13)
Productivity during Fellowship could have been better			
	Yes	41	(40)
	Maybe	33	(33)
	No	27	(27)
Fellowship fulfilled objectives			
	Yes	83	(82)
	Mostly	17	(17)
	No	1	(1)

Number of survey respondents per question: 101

Academic productivity during the fellowship was measured based on manuscript and abstract authorship. Fellows authored a median of 2 peer-reviewed manuscripts and 3 abstracts as first or co-author during their time in the program. A small group of individuals had obtained a Master’s (10%) or Doctoral (4%) degree during the program. Interestingly, 73% of respondents felt that their productivity could have been improved during the fellowship. Top factors limiting productivity (Table 7), in order of importance, included insufficient protected research time, limited collaborative opportunities with other fellows, mentorship/supervision, as well as challenges with research (e.g., lack of expertise and resources), and family/personal issues.

### Current Clinical and Scholarly Activities of Survey Respondents

A high percentage of respondents (68%) considered practicing in Canada after their fellowship; however, only 31% of alumni found employment in Canada immediately after graduating (Table 4). A small proportion of graduates (17%) enrolled in another fellowship or training program.

**Table 4:** Clinical and scholarly activities after Fellowship

		No.	Percent
Considered staying in Canada after Fellowship			
	Yes	69	(68)
	No	32	(32)
Country of employment immediately post-Fellowship (top 5)			
	Canada	31	(31)
	UK	20	(20)
	Australia	16	(16)
	Switzerland	6	(6)
	Chile	5	(5)
	Other	23	(22)
Enrolled in another fellowship or training program after UTDRO Fellowship			
	Yes	17	(17)
	No	84	(83)
Obtained additional qualifications since completing Fellowship			
	MBA	2	(2)
	MSc	9	(9)
	PhD	9	(9)
	None	81	(80)
Number of departments employed as staff oncologist after Fellowship			
	1	70	(69)
	2	28	(28)
	3	2	(2)
	4	1	(1)
Country of current employment (top 5)			
	Canada	30	(30)
	UK	19	(19)
	Australia	16	(16)
	Switzerland	6	(6)
	USA	5	(5)
	Other	25	(24)
Type of current practice			
	Community	5	(5)
	Mixed	18	(18)
	Private	5	(5)
	University affiliated	73	(72)
Type of current position			
	Academic	10	(10)
	Clinical	35	(35)
	Combined	56	(55)
Current title (top 5)			
	Consultant	33	(33)
	Assistant professor	22	(22)
	Associate professor	19	(19)
	Senior lecturer	10	(9)
	Professor	6	(9)
	Other	11	(11)
Held leadership position(s) post-Fellowship			
	Clinical/academic head of department	22	(22)
	Laboratory/research group leadership positions	22	(22)
	Management/administrative leadership position	62	(61)
Ongoing clinical or research collaborations resulting from Fellowship			
	Yes	47	(47)
	No	54	(53)
Number of peer-reviewed publications since leaving Fellowship			
	0	16	(16)
	1–5	40	(39)
	6–10	14	(14)
	11–15	5	(5)
	> 15	26	(26)

Number of survey respondents per question: 101

At the time of analysis, all respondents held staff positions in the field of radiation oncology and had been employed in a median of 1 department (range 1–4) as staff radiation oncologists since completing the fellowship. Most are currently employed in Canada (30%), UK (19%), or Australia (16%). The majority practiced at university-affiliated institutions (72%) in combined academic and clinical roles (55%); the top three occupations reported were consultant (33%), assistant professor (22%), and associate professor (19%). Respondents typically spent as a median: 60% (range 0–100%) of their time on direct patient care, 10% (range 0–70%) on research, 10% (range 0–70%) on administration, 10% on teaching/education (range 0–50%), and 0% (range 0–80%) on other duties. A large proportion (61%) had held management/administrative leadership positions since completing the fellowship.

Most respondents (39%) had authored 1–5 peer-reviewed publications since graduating, while 26% had published more than 15 papers. Approximately half (47%) had ongoing clinical or research collaborations originating from the fellowship; among which, approximately 70% (33/47) were with UTDRO faculty.

### Perceptions of Fellowship and Its Impact on Survey Respondents

Respondents identified acquiring clinical expertise, academic/research opportunities, supervision/mentorship, collegial and multidisciplinary environment, and networking opportunities (social/professional) as being the top five strengths of the UTDRO Fellowship Program (Table 7). Program weaknesses most commonly identified included heavy clinical load (e.g. on-call requirements), insufficient supervision/mentorship (e.g. research guidance, post-fellowship and career development advice, assistance with accommodations, family/personal issues), limited resources and time for research (Table 7).

Most respondents did not experience any financial stress (65%) or social challenges (74%) while participating in the program (Table 5). Among those who did report financial difficulty (35%), around 74% percent (26/35), had relocated to Toronto with their family. Higher costs of living in Toronto, lower fellowship stipends than in their home country, as well as other financial commitments (e.g., relocation costs, family/child care support, mortgages in home country) were common sources of financial stress among respondents (Table 7). The main social challenges experienced by individuals included having no social network support in Toronto, adjusting to a new city with a family, finding employment for spouse/partner, and communication barriers (Table 7).

**Table 5:** Perceptions of fellowship program and its impact

		No.	Percent
Overall experience as UTDRO Fellow			
	Far below expectations	0	(0)
	Barely met expectations	2	(2)
	Met expectations	6	(6)
	Above expectations	46	(45)
	Far above expectations	47	(47)
Impact of Fellowship on career development			
	Strongly negative	0	(0)
	Negative	0	(0)
	None	4	(4)
	Positive	37	(37)
	Strongly positive	60	(59)
Comparison with local radiation oncology peers			
	Less successful	0	(0)
	Slightly less successful	0	(0)
	No difference	26	(26)
	Slightly more successful	51	(50)
	More successful	24	(24)
Experienced financial difficulty during Fellowship			
	Yes	22	(22)
	Maybe	13	(13)
	No	66	(65)
Experienced social challenges coming to Toronto			
	Yes	26	(26)
	No	75	(74)
Would recommend the Fellowship Program to others			
	Yes	97	(96)
	Maybe	3	(3)
	No	1	(1)
Ongoing communication with other UTDRO Fellows			
	Yes	92	(91)
	No	9	(9)

Number of survey respondents per question: 101

Overall, the majority of respondents (92%) felt that the UTDRO Fellowship exceeded their expectations, wherein 99% of alumni would recommend the program to others. The vast majority (96%) indicated that completing the UTDRO Fellowship had a positive or strongly positive impact on their career development. In comparison with their local RO peers without UTDRO Fellowship training, 74% of alumni believed they were slightly or much more successful in their careers.

### Employment and Scholarly Activities of FRCPC Radiation Oncologists-UTDRO Fellowship Alumni Versus Others

Current employment and location status, as well as indicators for academic productivity, were identified for 285/287 of FRCPC designees in the comparator cohort (Table 6). Ninety-one percent of UTDRO Fellowship alumni and 89% of non-UTDRO peers were employed in Canada. All held staff positions in the field of Radiation Oncology. Many individuals from both groups held academic positions, in addition to clinical and/or research positions. University faculty appointments and research position(s) were held by 81% and 32% of UTDRO Fellowship alumni, respectively, compared with 68% and 10% for non-alumni peers. Leadership appointments were held by 26% of UTDRO Fellowship alumni vs. 17% of the non-UTDRO group; these positions included leads, heads, or directors of clinical, research, or educational programs, as well as leadership roles in regional, national, or international committees and organizations. University of Toronto, Department of Radiation Oncology Fellowship alumni had a higher median number of total publications (14.0 vs. 7.0; *P* = .04) and as first author (3.0 vs. 1.5; *P* = .002). There was no significant difference in the total number of clinical trials in which an individual was involved since receiving the FRCPC designation between both groups (*P* = .14).

**Table 6:** Employment and scholarly activities of FRCPC designees (2000–2012)

		UTDRO Fellow (*n* = 57)	Others (*n* = 228)	*P*
		No. (%)	No. (%)	
Country of employment	Abroad	5 (9)	25 (11)	
	Canada	52 (91)	203 (89)	
Appointment	Leadership	15 (26)	39 (17)	
	Research	18 (32)	22 (10)	
	Staff oncologist	57 (100)	228 (100)	
	University faculty	46 (81)	155 (68)	
		Median (range)	Median (range)	
Publications	First author	3.0 (0–18)	1.5 (0–41)	0.0023
	Senior author	1.0 (0–13)	0 (0–81)	0.1324
	Total	14.0 (0–124)	7.0 (0–271)	0.0415
Clinical trials	Principal investigator	0 (0–5)	0 (0–10)	0.6248
	Total	1.0 (0–5)	1.0 (0–11)	0.1380

## Discussion

This study represents the first formal assessment of the UTDRO Fellowship Program, and the collective opinions of its graduates on their training experiences and perceived impact on professional development. Based on the metrics evaluated in this study, the UTDRO Fellowship Program has been successful; the majority of graduates reported positive training experiences and benefits to scholarly output and professional development.

We reported that 68% of survey respondents considered practicing in Canada upon completion of fellowship training; 35% (24/69) of these individuals had also completed RO residency training in Canada. Only 31% of survey respondents were able to find employment in Canada immediately post-fellowship; not surprisingly, most of these individuals (71%; 22/31) had also completed their RO residency training in Canada. The vast majority returned to their home countries or relocated abroad. These findings are consistent with previous reports suggesting that most fellowship graduates practice in a different location from their fellowship training [9], and signifies a perceived shortage of Canadian employment opportunities for newly certified radiation oncologists, which may be a motivating factor for graduate emigration [6]. This perceived shortage of employment opportunities may have also served as motivation to pursue a fellowship in the first place. Respondents cited becoming more competitive in the job market as the fifth most listed reason to complete a fellowship, and without the potential presence of social desirability bias, this may have been reported even more frequently. Indeed, empirical studies have reported that the tight job market in Canada correlates with a spike in fellowship enrollment seen in the late 2000s/early 2010s [10]. Those graduating from residency programs during these surges may have needed a fellowship to act as a bridge until securing a job, or to improve their chances of obtaining a job.

Over the past decade, an upward trend in scholarly output has been observed among Radiation Oncology residents during training [11], possibly due to concerted efforts by RO residency programs to foster research opportunities and mentorship and increase protected research time [12, 13]. Fittingly, research output (e.g., publications, abstracts, grants, collaborations) was reported as one of the highest valued metrics denoting success of fellowship training by graduates surveyed in this study. Respondents reported high levels of academic productivity during their training, with more than 87% of trainees producing at least one manuscript and abstract as first or co-author (Table 3). A large proportion of respondents (65%) remained in academic practice post-fellowship and continued to develop robust research careers. Among radiation oncologists who received their FRCPC designation between 2000 and 2012, those with UTDRO Fellowship training were three times more likely than non-UTDRO peers to hold research appointments, as well as produce more publications. These findings may suggest that research productivity during fellowship training translates to research productivity later in one’s career.

Components of the Fellowship Program that could be improved included better training experience related to research, mentorship, fellowship expectations, and alumni engagement (Table 7). The desire for more protected research time, structured supervision of research projects, and clarity of available research projects were common sentiments expressed by the survey respondents. As a program aiming to foster research-oriented physicians, these are important factors to consider as the amount of dedicated research time allocated during training has been shown to be a key determinant of research output [12]. The Fellowship Program has also taken concrete steps to address the findings that 35% of fellows reported financial stress during their Fellowship. Over the duration of the Fellowship Program, remuneration has increased and our program now adheres to the Professional Association of Residents of Ontario guidelines [14] which ensures that our fellows receive competitive remuneration, commensurate with their experience, and based on university guidance of costs associated with participation in a fellowship program. We have also refined the information provided to our fellows, including practical advice on making the transition as smooth as possible and building a community among our fellows upon arrival. Our Chief Fellows are now tasked with an ambassador role, providing support and advice, as well as setting appropriate general expectations for incoming fellows.

**Table 7:** Key themes identified from open-ended survey responses

Fellowship objectives	1. Gain clinical experience in chosen area of interest 2. Exposure to research opportunities with potential to publish 3. Travel abroad to obtain another perspective of radiation oncology in a large academic department 4. Build collaborations with world-class academics 5. Obtain employment
Reasons Fellowship fulfilled objectives	1. Clinical experience 2. Research opportunities 3. Networking 4. Mentorship 5. Led to employment
Best aspects of Fellowship	1. Acquiring clinical expertise 2. Academic/research opportunities 3. Supervision/mentorship 4. Collegial and multidisciplinary environment 5. Networking (social and professional)
Worst aspects of Fellowship	1. Overwhelming clinical load 2. Lack of supervision/mentorship 3. Lack of academic/research opportunities 4. Limited resources and time for research 5. Lack of a collegial and multidisciplinary environment
Factors that hindered productivity during Fellowship	1. Insufficient protected research time 2. Limited collaborative work with other fellows 3. Inadequate mentorship or supervision 4. Limited opportunities 5. Challenges with research (e.g., lack of expertise and resources, time allocation) 6. Family or personal issues
Ways Fellowship Program can be improved	1. Better programmatic structure and mentorship for research a. More protected research time b. More research project supervision c. Involve fellows in research early during the Fellowship d. Increase opportunities to collaborate with other fellows and residents e. Improve access to research opportunities not linked to fellow’s clinical supervisor 2. Improve participation/dedication of mentors/supervisors and consistency of supervision/mentorship among fellows 3. Set clear objectives/expectations between fellow and supervisor(s) at start of Fellowship 4. Provide formal review of progress during Fellowship (e.g., at 4 months) to allow for adjustments and targeted actions 5. Provide support system for personal matters (e.g., child care, work-life balance) 6. Increase fellow stipends and funds to attend conferences
Reasons Fellowship was financially difficult	1. Higher costs of living in Toronto vs. home country 2. Fellowship stipend was lower than stipend/salary in home country or other local Fellowship Programs 3. Relocating with family (e.g., moving costs, supporting family, child care) 4. Financial commitments in home country (e.g., mortgage) 5. Visiting family who remained in home country
Social challenges experienced coming to Toronto	1. No social support network in Toronto 2. Adjusting to new city with family 3. Finding employment for spouse/partner 4. Communication barriers; English was second language for fellow/family 5. Winter season/cold weather

The nature of training provided through the UTDRO Fellowship is primarily the responsibility of the supervisors(s). As there was no explicit curriculum, respondents felt that the formalization of goals and expectations between the fellow and supervisor(s) at the beginning of the fellowship would augment the learning experience and academic productivity.

Respondents also viewed mentorship as crucial to their training and career development, with many reporting supervision/mentorship in the top 5 positive aspects of the fellowship, while it was also reported as one the top 5 worst aspects of fellowship. Many fellows found interactions with their supervisors/mentors to be very positive and helpful for their careers, but a small minority also felt that some supervisors/mentors could have been more available/appreciative. These limited negative experiences existed simultaneously with positive experiences; thus, fellows reported both. Many fellows requested greater faculty guidance in preparing for job readiness, work-life balance, and other issues that may arise during their fellowship. Improving the quality of mentoring may also help to alleviate the social challenges experienced by trainees during the fellowship.

Results of our study have highlighted the inherent value in garnering real-time feedback from presently enrolled fellows, as it facilitates our ability to be nimble and promptly responsive to fellows’ dynamic needs. As such, since the completion of this study, a formal mid-fellowship evaluation to assess trainees’ progress and satisfaction with the program has been implemented. Various strategies to maintain and engage our extensive alumni network, ranging from establishing a formal UTDRO alumni organization, newsletters, mentorship program, and alumni awards, have also been established or are being explored.

Additional ways our study findings have allowed for tangible improvements within our program include an increased focus on research productivity as an important deliverable both in terms of consolidating learning and facilitating career planning. Enriched educational offerings including research rounds, a journal club dedicated to fellows, and participation in the Strategic Training in Transdisciplinary Radiation Science for the twenty-first century program (STARS21; a competitive program for transdisciplinary training in research skills essential to conducting innovative research in radiation medicine) are now made available and participation is strongly encouraged.

The present study has several limitations that should be noted. The accuracy of the current employment and academic output data is limited by the content reported online at the time of the search. Furthermore, comparing the career success and academic productivity of UTDRO graduates with a non-UTDRO cohort was challenging, as no appropriate comparator group was readily available. Employing FRCPC designees who had not completed the UTDRO Fellowship as the comparator had limitations, since it was unknown as to whether those individuals had completed fellowship programs at other institutions or additional forms of post-residency training. The possibility of selection bias is also acknowledged. While the comparator cohort provides an important frame of reference, it is pertinent to note that those who enrolled in our department’s formal Fellowship Program may have been more academically inclined prior to the start of the fellowship, and thus may have displayed higher levels research productivity regardless of the Fellowship Program itself. The potential benefits of completing a UTDRO Fellowship versus other forms of post-residency training or no training at all can only be inferred from these findings, warranting further investigation.

Another recognized constraint of our study is the self-reporting nature of the survey as it may be subject to recall and responder bias. Difficulty in eliciting physician participation in survey research has long been recognized, primarily due to demands on physicians’ time and resources. This is reflected in our study’s response rate of 38%, which remains within the range of those reported in similar surveys, varying between 12 and 57% [4, 15–17].

Additional survey questions that describe the broader impact of the Fellowship Program on employment, such as time to secure employment, number of job applications/interviews before accepting a job offer, and extent of knowledge transfer to a fellow’s home country, especially for fellows from LMICs where there is an explicit goal to enhance radiation oncology capacity building and knowledge transfer, merit further consideration. Subsequent evaluations could also be conducted on a regular basis, with supervising faculty being surveyed for a 360-degree assessment of the program.

In conclusion, our study demonstrates the perceived benefits of completing post-residency fellowship training, which may be becoming an expected extension beyond residency for graduates in some North American areas [2]. It highlights potential factors that appear to be most important to those pursuing fellowships, which can help inform academic program directors on strategies to improve recruitment and the overall training experience. We recognize that our findings may not be representative of all Canadian Radiation Oncology fellowship graduates, but we hope that these data will provide a framework for similar programs looking to evaluate and improve their curricula.

## Electronic supplementary material

ESM 1 (DOCX 580 kb)
