# Supplementary material for: Radiation Oncology Fellowship: a Value-Based Assessment Among Graduates of a Mature Program
Source: J Cancer Educ. 2020 Jul 18;36(6):1295–305. doi: 10.1007/s13187-020-01767-5 (PMC8605971; doi:10.1007/s13187-020-01767-5)

# Supplementary Material

**Appendix I: Study design**


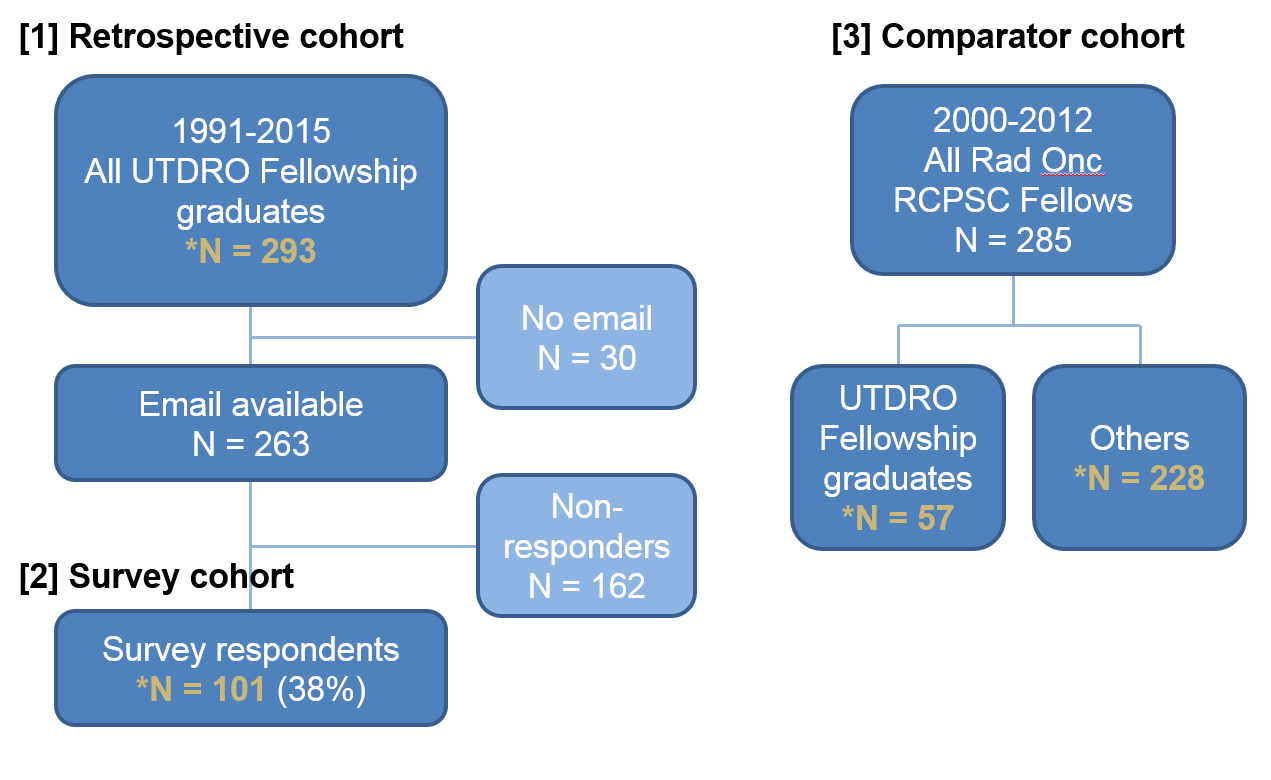


**Appendix II: Search strategy for academic productivity**

Academic productivity for the retrospective and comparator cohorts was determined using three strategies: (1) a search of cited peer-reviewed publications using databases from the National Center for Biotechnology Information (NCBI) (PubMed, PubMed Commons), Google Scholar, ResearchGate, ScienceDirect, Web of Science, Scopus-Elsevier and ORCID (UberResearch/UberWizard); (2) a search for registered clinical trial activity using the NIH U.S. National Library of Medicine ClinicalTrials.gov database; and (3) a detailed Google web search assigning specific sites and terms.

The first two strategies utilized site-specific search tools and strings. Each set of search strings was modified for the site’s search engine. For NCBI, the following search strings and tags were used: surname given name, surname first initial, “surname given name”, “surname first initial”, surname first initial[au]/[author], “surname first initial”[au]/[author], surname given name[au]/[author], “surname given name”[au]/[author]. Each of these search strings was then repeated adding the location, either through the ‘AND’ function or exact phrase (e.g. AND Toronto or “Toronto”). Google Scholar, ResearchGate, and ClinicalTrials.gov searches utilized a general, wild-card, and exact-phrase searching method. The general search retrieved results using the surname, surname + given name, surname + first initial. Wild-card searches truncated the given names after the first initial to identify variable spellings and formats of given names; if this yielded irrelevant results, hyphenated surnames were truncated after the last letter before first hyphenation. Exact-phrase searches produced results that exactly matched the last record we had of the name in question. ScienceDirect, Web of Science, Scopus-Elsevier, and ORCID searches made use of the site-specific tools, which allowed for field searching by author, institution/affiliation, and unique identifiers that generated the data for academic productivity.

The Google web search engine provided a more general and encompassing approach to determining academic productivity. Searches were conducted using a specific set of terms coupled with the name in different combinations (surname, surname + given name, surname + first initial) and last known location (L). The following strings were most commonly used:

(1): name combination “radiation oncology”;

(2): name combination “radiation”;

(3): (1) or (2) (L);

(4): (1) or (2) email address;

(5): “surname given name”;

(6): “surname” wild-card first initial,

(7): (5) or (6) radiation;

(8): (5) or (6) radiation oncology;

(9): (5) or (6) “radiation”;

(10): (5) or (6) “radiation oncology”.

This approach also searched the abovementioned databases that were indexed by Google. Strings utilized in this method included:

(1): site:indexed site (e.g. site:www.clinicaltrials.gov) name combination;

(2): “indexed site” (e.g. “www.clinicaltrials.gov”) name combination;

(3): site:indexed site “surname given name”;

(4): “indexed site” “surname given name”;

(5): site:indexed site “surname” wild-card first initial.

**Appendix III: Origin countries of UTDRO Fellows**


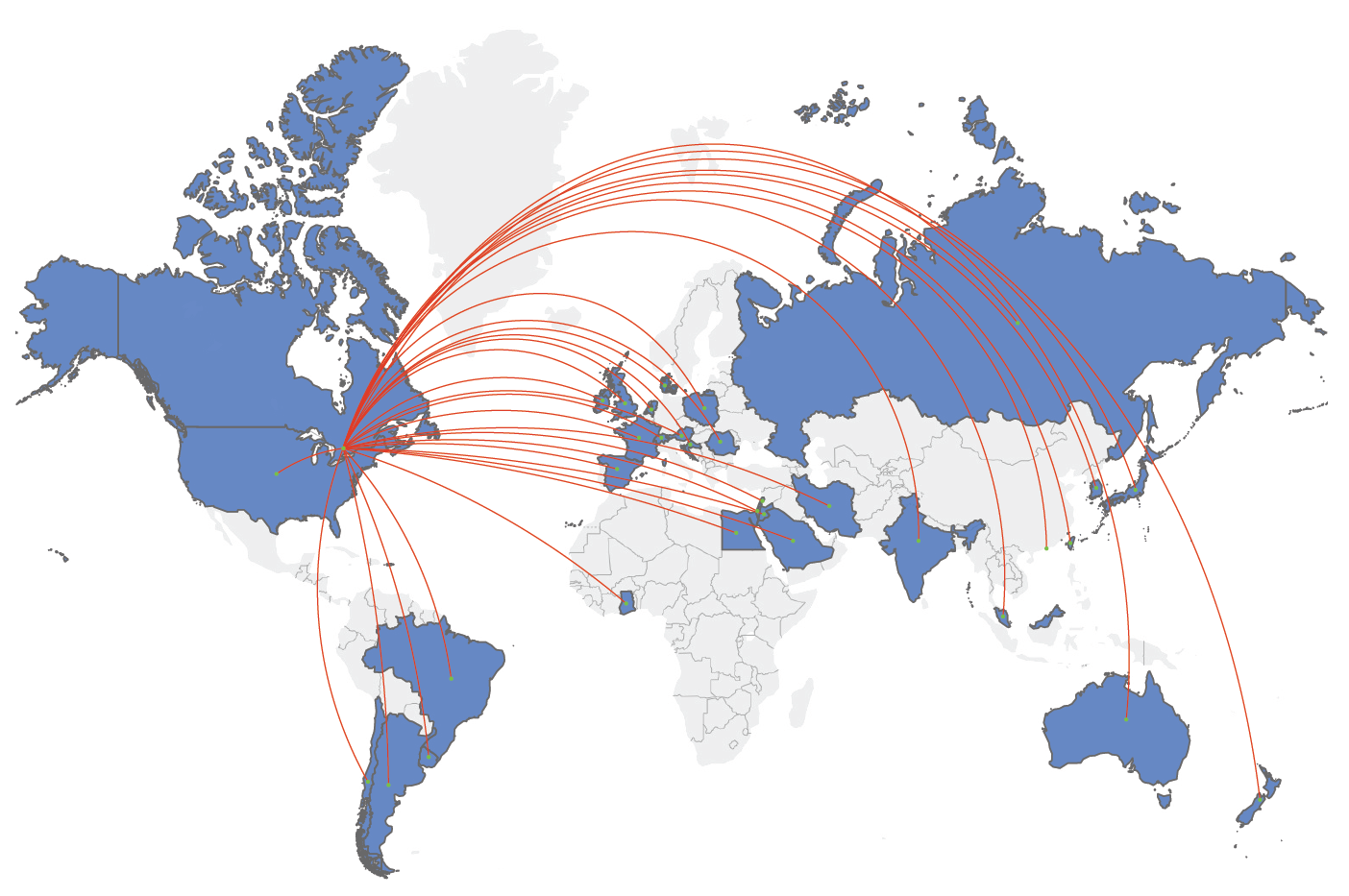

Supplement: Supplementary file 1 — (DOCX 580 kb) [file 13187_2020_1767_MOESM1_ESM.docx]
